# Optical, Mechanical, and Chemical Impact of Brushing with Activated Charcoal Toothpowder and Toothpaste on Dental Enamel: An In Vitro Evaluation

**DOI:** 10.3390/ma17246104

**Published:** 2024-12-13

**Authors:** Eva Aline Costa Cutrim, Karla Janilee de Souza Penha, Patrícia Barbosa da Silva, Edilausson Moreno Carvalho, Mayron Guedes Silva, Cristie Luis Kugelmeier, Leily Macedo Firoozmand

**Affiliations:** 1 Department of Dentistry, Federal University of Maranhão (UFMA), São Luís 65080-805, MA, Brazil; eva.aline@discente.ufma.br (E.A.C.C.); patricia.bs@discente.ufma.br (P.B.d.S.); 2 Postgraduate Program in Dentistry, Federal University of Maranhão (UFMA), São Luís 65080-805, MA, Brazil; karlajanilee@gmail.com (K.J.d.S.P.); mayron.guedes@discente.ufma.br (M.G.S.); 3 Postgraduate Program in Dentistry, CEUMA University, São Luís 65075-120, MA, Brazil; edilausson@gmail.com; 4 Department of Materials Engineering, Federal University of São Carlos (UFSCar), São Carlos 13565-905, SP, Brazil; cristieluis@gmail.com

**Keywords:** dental enamel, brushing, microhardness, roughness, ion release

## Abstract

This in vitro study evaluated the effects of brushing with activated charcoal powder or toothpaste on enamel surface properties, including color change (ΔE), Knoop microhardness (HK), roughness (Ra), and the characteristics of the resulting brushing slurry [pH, fluoride (F^−^), and calcium (Ca^2+^) concentration]. A total of 48 enamel samples were stained and divided into 4 groups (*n* = 12): activated charcoal toothpaste (AC-T), activated charcoal powder (AC-P), hydrogen peroxide-based whitening toothpaste (HP-T), and conventional toothpaste (C-T, positive control). The samples were subjected to a brushing cycling model, and ΔE, HK, Ra and enamel morphology were analyzed at baseline (T0) and after brushing cycle (T1). Additionally, the pH, soluble F^−^, and Ca^2+^ concentrations of the slurry were analyzed. Data were analyzed using ANOVA/Tukey and paired *t*-tests (α = 0.05). After brushing, AC-T and AC-P (*p* < 0.05) showed a decrease in HK, an increase in Ra, and no ΔE variation was observed between groups (*p* = 0.163). All products had alkaline slurries (>7), and AC-P had no detectable F^−^ (*p* = 0.00) with significantly higher calcium loss (*p* = 0.015). Fewer enamel topographical changes were observed in C-T. In conclusion, brushing with CA toothpowder and toothpaste does not promote color change, but toothpowder increases surface Ra, decreases enamel HK, lacks F^−^, and causes greater Ca^2+^ loss.

## 1. Introduction

Daily toothbrushing is considered one of the most important methods for maintaining oral health [1,2]. The pathogenic nature of plaque biofilm can be reduced by decreasing the bioburden through dynamic biofilm control [2]. It is therefore important to control dental dysbiosis, which represents an imbalance in the microbial community [3], so that it does not lead to oral health problems such as caries, periodontal disease, and other oral infections. Toothpaste has been proven to be an adjunct to the brushing technique and can be used as a vehicle for therapeutic agents that help improve oral health [4].

More than just cosmetic products, toothpastes contain therapeutic and abrasive agents that facilitate biofilm removal [4]. Considering these aspects, fluoride (F^−^) has been the most important therapeutic agent incorporated into toothpastes, with strong evidence showing its ability to reduce caries in children and adults when used at a minimum concentration of 1000 ppm and a frequency of at least twice a day [4].

On the other hand, abrasives are considered to be important components for the removal of dental pigmentation [5]. The American Dental Association (ADA) recommends that the abrasiveness of toothpastes should not exceed 40 REA (Relative Enamel Abrasion) and 250 RDA (Relative Dentine Abrasion) [6] to avoid potential irreversible damage to tooth structure [7]. However, driven by the constant aesthetic appeal for ever-brighter teeth, household products such as whitening toothpastes have become prominent on the market [8]. Although abrasives are potentially capable of causing irreversible structural damage to enamel [9], they are the main functional ingredient in whitening toothpastes, which often contain higher levels of abrasives and detergents compared to conventional toothpastes [8].

Within this context, activated charcoal (AC) has been incorporated into whitening toothpastes in order to remove and prevent tooth stains [10]. AC is produced by heating carbonaceous materials in association with substances capable of producing porous internal surfaces, thereby ensuring their activation [11]. Its chemical action is believed to be due to its ability to adsorb chomophores and extrinsic pigments due to its high surface area [12]. Although AC has been shown to be beneficial in several health areas, such as body detoxification and water purification [13], it does not appear to have this effect on dental enamel [9]. Furthermore, the mechanical effect of AC particles has been questioned [14], as AC may have a higher abrasive potential during tooth brushing, potentially leading to irreversible loss of tooth enamel [15].

Although the relative dentine abrasion (RDA) and relative enamel abrasion (REA) values of some charcoal toothpastes appear to be similar to those of commercially available toothpastes [16], chemical aspects, such as the profile of the amount of soluble F^−^ and Ca^2+^ ions following the use of AC-containing products in the oral environment, remain underexplored in the literature [17]. There is evidence that pH changes and the presence of these ions could influence enamel behavior under continuous erosive challenges [15]. However, to date, no studies have reported on the interaction between the physico-mechanical properties of dental tissues and the chemical aspects of the application of AC-containing agents. In addition, the lack of fluoride compounds in many of the charcoal-based toothpastes studied may reduce their protective benefits for consumers, highlighting the importance of educating patients accordingly [16]. Thus, the novelty of this study is to elucidate the mechanical and physical effects of brushing with AC-based products, along with the chemical characteristics of the resulting slurry.

Therefore, the aim of this study was to evaluate, in vitro, the influence of toothbrushing with activated charcoal (AC) (powder or dentifrice) on the physico-mechanical properties (color change (ΔE), microhardness (HK) and roughness (Ra) of the tooth structure), as well as the chemical properties [pH change, presence of fluoride (F^−^) and calcium (Ca^2+^) ions] of the solutions generated after toothbrushing. The null hypotheses tested were as follows: (1) there is no change in HK and Ra after brushing, (2) there is no change in enamel color with the use of AC in toothpowder or toothpaste, (3) there is no difference in the pH of the solutions obtained after brushing, and (4) there is no difference in the concentration of soluble F^−^ and Ca^2+^ ions in the solutions.

## 2. Materials and Methods

### 2.1. Study Design

This randomized, controlled, double-blind, in vitro study used bovine enamel samples to compare the surface microhardness (HK), surface roughness (Ra) and color change (ΔE) of bovine enamel brushed with different whitening toothpastes and toothpowders. The whitening toothpastes used in this study were grouped according to their active whitening mechanism: activated charcoal (powder), activated charcoal (toothpaste), active hydrogen peroxide (toothpaste), regular toothpaste. This study followed a 4 × 2 factorial design with the following experimental factors: treatments (4 toothpastes) and 2 levels (before and after brushing). The response variables were surface microhardness (HK), surface roughness (Ra), color change (ΔE). Additionally, pH, and F^−^ and Ca^2+^ concentrations in the solutions produced by these brushes were also analyzed. The primary outcome was a change in tooth color after brushing and the secondary outcomes included the other measured variables.

### 2.2. Sample Size Calculation

For sample size determination, the primary outcome (color change) was considered in the calculation. For calibration, 2 color evaluations of 20 teeth were performed 7 days apart, yielding a Kappa value of 0.647 [18], with the evaluator blinded to the experimental group. The non-inferiority sample calculation was carried out using G*Power 3.1 software (Francz Faul Universitat, Kiel, Germany), based on pilot test values. An ANOVA test was applied, considering a 95% confidence interval, a significance level of 0.05 with 80% of the study’s testing power. Considering a potential sample loss of 20%, a total of 48 samples were used, divided into 4 groups (*n* = 12).

### 2.3. Sample Preparation

Freshly extracted bovine incisors, free from cracks and caries, were used. The teeth were sectioned 2 mm below the amelocemental junction [19] and the roots were discarded. The crown was cleaned with pumice paste and water using a Robinson brush (KG Sorensen, São Paulo, Brazil) at low rotation. The pulp chamber was cleaned with curettes and air-water jets.

**Figure 1:**
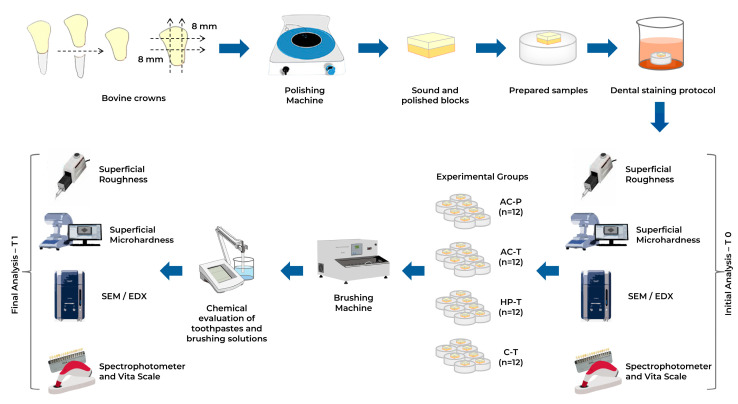
Experimental study design. Bovine crowns were prepared and subjected to a dental staining protocol. Samples were divided into four groups and treated with specific solutions using a brushing machine. Initial (T0) and final (T1) analyses were performed to evaluate the effects of treatment.

The buccal surface was sectioned with a cutting machine (Isomet 1000, Buehler Lake Bluff, IL, USA) to obtain samples measuring approximately 8 mm × 8 mm (Figure 1). The lingual surface was removed and discarded, and only the buccal surface was retained for analysis [19]. The enamel was ground using a metallographic polisher (AROPOL, Arotec, Cotia, Brazil) with silicon carbide grinding papers (#600, #800, #1200 and #2500) to flatten the buccal surface and obtain flat and smooth enamel surfaces. The final sample’s thickness was 3 mm [20], consisting of 1 mm of enamel and 2 mm of dentin [21]. The sample dimensions were checked using a digital caliper (Absolute Digimatic, Mitutoyo Sul Americana Ltda. São Paulo, Brazil). 

### 2.4. Dental Staining

After polishing, only the dentin portion of the samples was etched with 37% phosphoric acid gel for 15 s, followed by washing with water and air jets for 30 s [19]. This dentinal surface procedure was performed to remove initial stains and open the dentinal tubules to allow the pigment solution to penetrate the surface [22]. To standardize the initial color of the teeth, the samples were embedded in resin blocks, leaving only the enamel exposed.

The tooth staining protocol was performed as described in the literature [22]. A tea solution was prepared with black tea (Chá Leão, Fazenda Rio Grande, PA, Brazil) using 2 g of tea per 100 mL of distilled water, boiled for 5 min, and filtered to remove the tea from the infusion. All samples were immersed in the tea solution to standardize the initial tooth color. The tea solution was renewed daily, and the samples were stored and monitored at room temperature for six days [22].

After this period, the samples were rinsed in distilled water and submitted for color measurement (Figure 1).

### 2.5. Enamel Color, Microhardness, and Surface Roughness

The initial color was recorded using the Vita Classical scale and the Easyshade spectrophotometer (Vita Zahnfabrik, Bad Sackingen, Germany), with its tip positioned at a 90° angle to the buccal side of the sample [19]. To ensure accurate measurements, the samples were placed on a white background [23], with a circular protection made of opaque material to prevent external light from passing through and interfering with the results [24].

Upon completion of the tests, the data were presented as ΔL*, Δa*, and Δb* [24], where ΔL* denotes the variation in luminosity (ranging from 0 to 100), Δa* represents the variation along the blue/yellow axis, and Δb* describes the variation along the red/green axis [25]. Color variation before and after brushing was recorded as delta E (ΔE), calculated according to Equation (1):

ΔE = [(ΔL*)^2^ + (Δa*)^2^ + (Δb*)^2^]^1/2^ (1)

Initial evaluations of Knoop microhardness (HK) and surface roughness (Ra) were performed at two experimental times: (T0) before the experimental groups were divided, and (T1) after the brushing protocol (Figure 1). The HK assessment was performed using a microhardness tester (HMV-G20, Shimadzu-Future-Tech Corporation, Tokyo, Japan). Three indentations were made in the central area of each sample at 500 µm intervals between each measurement, and the average microhardness value for each sample was obtained from these indentations. A force of 980 g was applied for 15 s [26]. Surface roughness (Ra) was measured using a digital roughness meter (Mitutoyo SJ-210, code 178-561-02A, Sakado, Japan). The roughness assessment was based on the arithmetic mean of valleys and peaks recorded by the device, with the result represented by the Ra unit [21]. Measurements were taken from the center, left, and right regions of each sample. The force applied by the needle was 5 N, with an average speed of 0.05 mm/s over a measurement distance of 2.5 mm [24].

### 2.6. Experimental Groups

After the initial HK readings and color measurement of the samples, they were randomized [21] and stratified into groups (*n* = 12) using the web tool Random.org (www.random.org, accessed on 23 March 2023). The groups were defined according to the treatments: **AC-T**—Activated charcoal-Toothpaste; **AC-P**—Activated charcoal-Powder; **HP-T**—Toothpaste with hydrogen peroxide; **C-T**—Control-Toothpaste. The experimental groups and the products tested are described in Table 1.

The slurries of activated charcoal and other toothpastes were prepared in a 1:3 (*w*/*w*) ratio of the product to distilled water [27], and the pH was measured. A selective F^−^ ion electrode (model 18AF, Analyser, São Paulo, SP, Brazil) coupled to a digital pH/F^−^ analyzer (Quimis, model Q400ISE, Diadema, SP, Brazil) was used, after calibration with standard solutions [28]. Twelve measurements were taken, and the mean value was used as the final pH.

### 2.7. Brushing Protocol

For the brushing protocol, the samples were positioned in a mold, and the enamel was brushed with the slurries using soft bristle brushes (C. KOVACS, São Paulo, Brazil), fixed to the brushing machine by a 7.8 mm diameter handle.

Dental brushing was performed using a brushing machine (model P-200, Biopdi, São Carlos, SP, Brazil), which consists of a brushing simulation machine that allowed simultaneous brushing of 10 specimens. This brushing machine reproduces a linear movement and allows the programming and adjustment of weight, speed and number of cycles to standardize the application of force, intensity and test time. Thus, according to the literature, a load of 4 N was programmed [23], with 108 movements per cycle, and each brushing cycle was performed at 0.6 Hz (36 cycles/min) for three minutes, reproducing a total of 3240 strokes [23,29]. Throughout the brushing process, the samples were soaked in a solution consisting of a mixture of activated charcoal powder or toothpaste and distilled water, with each component added in a 1:3 ratio [23,27,29]. After the brushing cycles were completed, the solution from the brushes was collected using millimeter syringes and stored in sterile plastic vials. The brushed samples were washed in running water and dried with air jets.

### 2.8. Scanning Electron Microscopy (SEM) Characterization: Morphology of Charcoal Powder Particles and Enamel Surface

Images of the activated charcoal powder and the enamel surface, before and after abrasion, were obtained. The surface morphology and microstructural characterization of the charcoal powder particles and enamel surface were characterized using a field emission scanning electron microscope (SEM) (Hitachi TM 3030, Hokkaido, Japan), and elemental analysis was performed using the energy-dispersive X-ray spectroscopy (EDX) mode in the SEM. To evaluate the activated charcoal powder particles, a set of samples was prepared by evenly depositing dry powder on carbon tape attached to a SEM stub. Photomicrographs of the activated charcoal powder particles were taken at 200× and ×1000 magnification.

Three enamel samples from each experimental group were selected, dried overnight in an oven and mounted on aluminum stubs with carbon adhesive tape (Koch, Instrum Cient, São Paulo, SP, Brazil) for qualitative surface morphology evaluation in SEM. Images were acquired with the aforementioned SEM, which allowed analysis of enamel morphology at a magnification of ×5000.

### 2.9. Chemical Solution Evaluation: pH and Fluoride/Calcium Ion Release

After the brushing cycle protocol (Figure 1), 10 mL of the solution from each experimental group was collected and stored in plastic flasks. Before measuring the fluoride (F^−^) and calcium (Ca^2+^) concentrations, 5 mL of the solution was used to check the pH, using a pH electrode (Quimis, model QA 338 ECV, Diadema, SP, Brazil) connected to a pH/F^−^ digital analyzer (Quimis, model Q400ISE, Diadema, SP, Brazil) [28].

### 2.10. Verification of Ionic Release: Fluoride and Calcium

To determine the initial concentrations of fluoride (F^−^) and calcium (Ca^2+^) ions, the solutions prepared with toothpaste/powder and distilled water were measured before brushing. After brushing, new measurements were carried out to determine the F^−^ and Ca^2+^ concentrations present after the mechanical action of the toothbrush with the abrasives on the enamel. The same concentrations of toothpowder/paste and distilled water were standardized in the solutions analyzed to obtain consistent measurements before and after brushing.

The concentration of F^−^ in toothpaste can be determined by measuring the fluoride ion (FI), total soluble F^−^ (TSF) [the sum of FI and fluoride as sodium monofluorophosphate, Na_2_FPO_3_, (MFP)] and total fluoride (TF) (the sum of total soluble fluoride (TSF) and insoluble F (IF) which is the F bound to the abrasive). Thus, from the solution generated after brushing, 5 mL of solution from each brushed sample was separated and the total soluble fluoride (TSF) ion content was measured using a selective F^−^ ion electrode (model 18AF, Analyser, São Paulo, SP, Brazil) coupled to a digital pH/F^−^ analyzer (Quimis, model Q400ISE, Diadema, SP, Brazil) [28].

To assess total soluble fluoride (TSF), a 1:3 dilution was made, and triplicates of 0.25 mL of the suspension were transferred to test tubes. The remainder of the suspension was centrifuged (3 g, 10 min, room temperature) to remove the insoluble fluoride bound to the abrasive [30,31]. Triplicates of 0.25 mL of the supernatant were transferred to test tubes to determine the TSF concentration (sum of fluoride as F^−^ and MFP ions).

For all TSF tubes, 0.25 mL of 2.0 M HCl was added and, after 1 h at 45 °C, the samples were neutralized with 0.5 mL of 1.0 M NaOH and buffered with 1.0 mL of TISAB II (1.0 M acetate buffer, pH 5.0, containing 1.0 M NaCl and 0.4% CDTA) [30,31]. The F^−^ ion selective electrode, coupled to a digital pH/F^−^ analyzer, was previously calibrated with fluoride standards (final F^−^ concentration: 0.625; 1.25; 2.5; 5; 10 and 20 ppm F^−^), prepared with the same reagents as the sample. A 0 ppm F^−^ standard was also prepared as a control. For the experimental group analysis, a linear equation was calculated correlating the logarithm of the F^−^ concentration in the standards and mV. Microsoft Excel software, version 2410 (Microsoft Corp., Redmond, WA, USA), was used to determine the concentration of F^−^ in each toothpaste, expressed in ppm (μg F/g).

Calcium analyses of enamel brushed with different toothpaste whitening mechanisms were performed by using spectrophotometry. To determine the Ca^2+^ ion concentrations, 5 mL of the solution from each of the twelve samples in each experimental group was evaluated. The solutions were analyzed by Inductively Coupled Plasma Optical Emission Spectrometry (ICP-OES-9820; Shimadzu, Kyoto, Japan) [28]. The test was performed in triplicate, and the values were measured in mg/L.

The Arsenazo III Calcium kit (Bioclin, Quibasa-Quimica, Belo Horizonte, MG, Brazil) was used as a colorimetric reagent [28,32,33]. A quartz micropipette of approximately 10 μL was used to provide standardized volumes of calcium standards. Using a microplate reader (Biotek Eon, Winooski, VT, USA), 1 mL of reagent 1 (standard) without Ca was added to each well of the plate. In another well, 1 mL of the standard reagent and 10 μL of reagent 2 (calcium carbonate, CaCO) were added.

To read the samples, 1 mL of Reagent 1 (standard) and another 10 μL of the solution from each experimental group were added in triplicate. The plates were shaken for 60 s in the microplate reader to allow the reaction between the sample and Arsenazo III. To obtain the absorbance result, the microplate was placed in the BioTek spectrophotometer (ELx800tm, Winooski, VT, USA). Absorbance readings were taken at 630 nm, and Ca^2+^ concentrations were calculated using Microsoft Excel software, version 2410 (Microsoft Corp., Redmond, WA, USA).

After measuring the Ca^2+^ concentration, the Ca^2+^ content of the solution was checked by subtracting the Ca concentration of the product before brushing (T0) and after brushing (T1), using the Equation (2):

Calcium lost to the environment = [Ca^2+^]_T0_ − [Ca^2+^]_T1_ (2)

### 2.11. Statistical Analysis

The data for ΔE, HK, Ra, pH, F^−^ and Ca^2+^ ion release were analyzed for normality and homoscedasticity using the Shapiro–Wilk test. The values obtained were subjected to repeated measures analysis of variance (ANOVA) with Bonferroni correction, and post-hoc Tukey test was applied. The variation factors were (1) whitening toothpaste mechanism, and (2) the time of evaluation (before and after). *t*-test was used to confirm the behavior of the enamel (microhardness and roughness) with the same toothpaste before and after brushing. The SPSS Statistical Software, version 26.0, (IBM Corp., Armonk, NY, USA) was used with a significance level of 5%.

## 3. Results

### 3.1. Surface Microhardness and Roughness of Enamel

The comparison of microhardness values among the toothpastes revealed that AC-T (*p* = 0.001) and AC-P (*p* = 0.005), activated charcoal-based products, caused a decrease in enamel microhardness compared to baseline (before brushing, T0) (Table 2). The initial microhardness values of the enamel surfaces (T0) were standardized for all the groups studied (*p* = 0.069).

Similarly, the initial roughness (T0) of all groups was standardized (*p* = 0.183). After brushing with the toothpastes, all enamel surfaces showed a significant increase in roughness (*p* < 0.05). The smallest change in roughness (Ra) was observed for C-T, while the highest change was observed for HP-T (*p* < 0.001) (Table 2).

### 3.2. Color Stability Evaluation

The mean, standard deviation and confidence interval of ΔE calculated after brushing for the experimental groups are shown in Table 3. No statistical difference between the toothpastes/toothpowder in terms of color variation was observed (*p* = 0.676).

### 3.3. Chemical Evaluation of Slurries: pH Measurements

**Figure 2:**
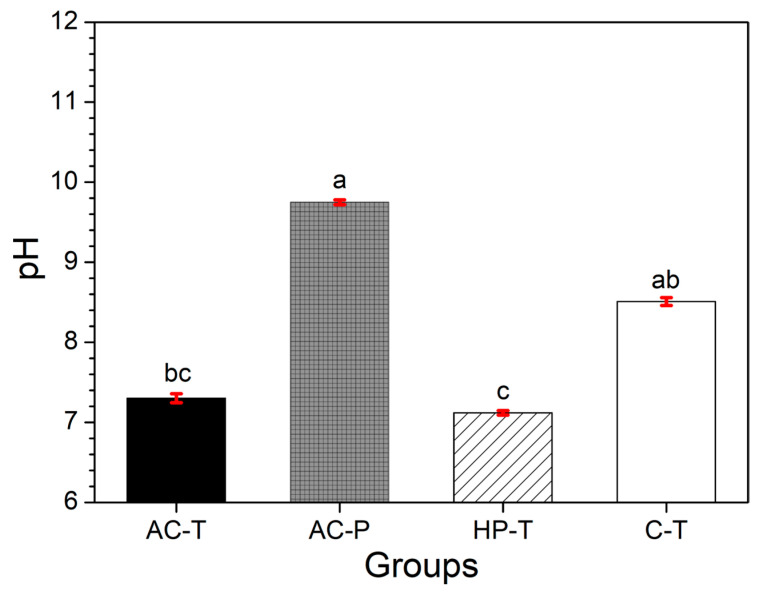
Mean values and in red standard deviation (SD) of solution: pH after brushing (different letters: *p* < 0.001).

The pH values of the brushing solutions are shown in Figure 2. The pH values of the solutions were found to be alkaline in all groups; with the AC-P and C-T groups having pH values higher than 9.

### 3.4. Chemical Evaluation of Slurries: Soluble Fluoride and Calcium Ions

**Figure 3:**
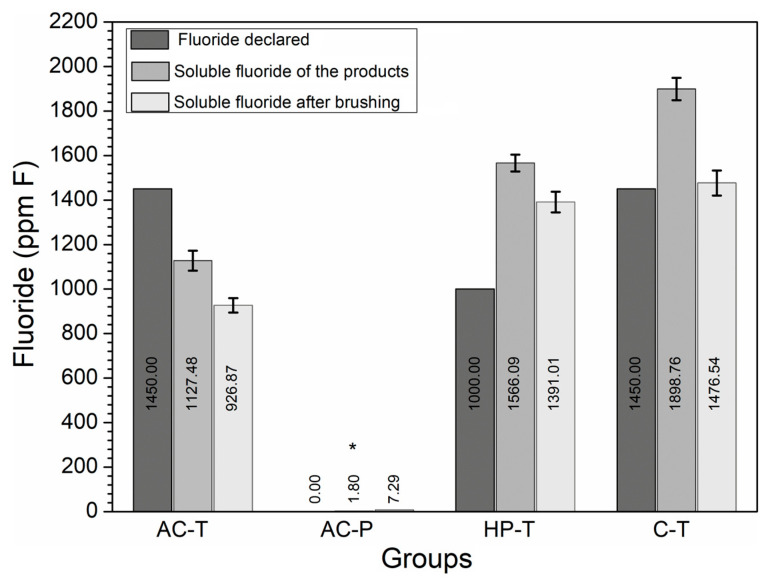
Concentration of fluoride (ppm F^−^) declared by the manufacturer (F declared), total soluble fluoride (SF) of the products (T0) and SF present in the solution after brushing (T1). (*) The presence of an asterisk indicates a statistically significant difference (*p* = 0.02).

The soluble F^−^ concentrations in the solutions obtained from brushing with the products are shown in Figure 3. It can be seen that all the groups had F^−^ values greater than 1000 ppm, except for AC-P (*p* = 0.02).

**Figure 4:**
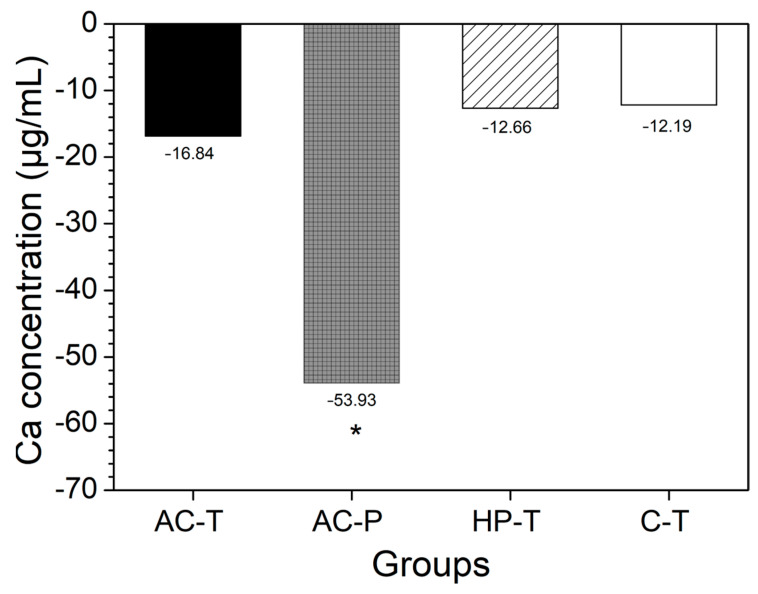
Difference in mean values of calcium concentrations (μg/mL) in the solution before (T0) and after the brushing protocol (T1) for each experimental group (*p* < 0.05). (*) The presence of an asterisk indicates a statistically significant difference.

Among the toothpastes used, the C-T group had the highest mean values (SD) of free calcium equivalent with 85.22 (10.39), while AC-T had 34.16 (27.32) and HP-T 27.50 (17.89). However, when the difference in concentration was calculated [according to Equation (2)], negative values were observed, indicating a loss of Ca^2+^ to the medium. The solution from brushing with activated charcoal powder (AC-P) showed a higher concentration of Ca^2+^ ions (*p* = 0.015), while no significant difference was found in the other groups when comparing the difference in Ca^2+^ ion concentrations between T0 and T1 (Figure 4).

### 3.5. SEM Results of Charcoal Powder Particles and Enamel Surface Morphology

**Figure 5:**
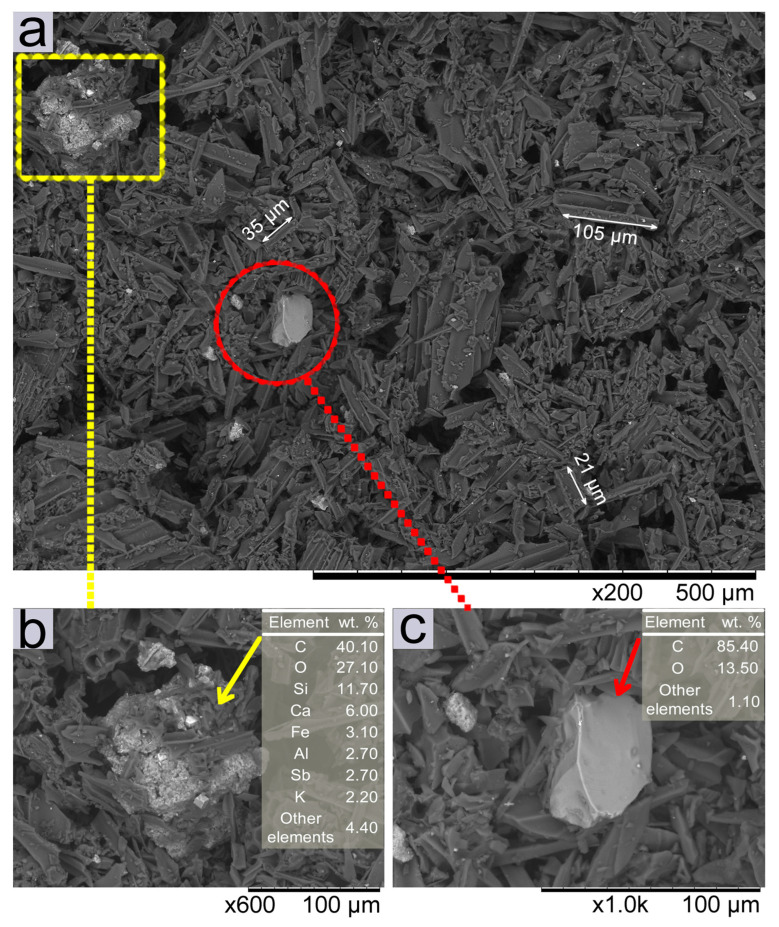
BSE-SEM images of activated charcoal powder (AC-P) showing (**a**) an overview of the charcoal sample with different particle sizes and agglomerates, (**b**) ×600 magnification detailing the morphology of the agglomerate and the table with EDS chemical analysis, and (**c**) ×1000 magnification detailing the particle morphology and the table with EDX chemical analysis. The arrows (→) indicate the particles on which chemical analyses by EDS were performed.

The microstructure and morphology of the activated charcoal powder were analyzed by backscattered electron (BSE) SEM images at ×200, ×600, and ×1000 magnifications (Figure 5). The chemical contrast between the different particles that make up the product (activated charcoal) was also analyzed [Figure 5a–c]. The AC-P consists of irregular particles (dark gray regions), with structural and morphological analysis revealing longitudinally fragmented charcoal microtubes of different sizes [Figure 5a]. In addition, some inhomogeneous agglomerates were noted [as shown in detail in Figure 5b], with different sizes and shapes (light gray regions) from those observed in this powder. A broad distribution of particle sizes was observed, ranging from less than 1 µm to more than 100 µm.

EDX chemical composition analyses [Tables inserted in Figure 5b,c] show that, as observed at ×600 magnification, the particle observed in Figure 5b, is an agglomerate composed mainly of C and O, with the presence of Si, Ca, and Fe, and other elements in lesser amounts. The compositional profile, as observed in the EDX analysis, indicates the presence of impurities in the charcoal powder, with a significant amount of Ca (6.0%), among other components [Figure 5b]. It is also noted that the agglomerate exhibits an irregular morphology, and the impregnation of charcoal powder particles on its surface (dark gray color). On the other hand, Figure 5c, at ×1000 magnification, shows a more homogeneous particle (light gray color), predominantly composed of C and O (around 85 and 13%, respectively), and other elements in lower concentrations, surrounded by other smaller charcoal powder particles.

**Figure 6:**
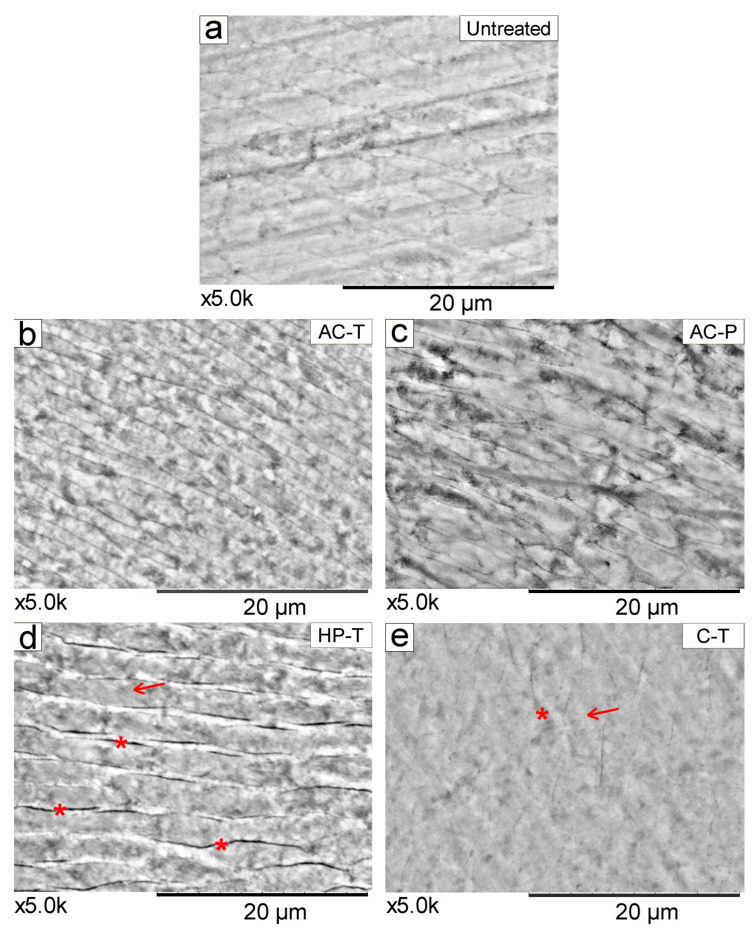
SEM images (×5000 magnification) of the tooth showing the morphology with enamel-like crystals in the different experimental conditions. In (**a**) sound tooth enamel (before brushing), (**b**) activated charcoal-toothpaste (AC-T), (**c**) activated charcoal-powder (AC-P), (**d**) hydrogen peroxide-toothpaste (HP-T), and (**e**) control-toothpaste (C-T). The arrows (→) indicate the rods in the enamel. (*) Visualization of interprismatic spaces, which are more evident in HP-T, compared to the other groups.

Representative SEM images of untreated tooth enamel at ×5000 magnification before (T0) [Figure 6a] and after brushing with different products (T1) [Figure 6b–e] are shown. Both the untreated enamel Figure 6a and enamel brushed with the control toothpaste Figure 6e show a more homogeneous topographical pattern of rods and inter-rods. In contrast, tooth surfaces brushed with HP-T show more evidence of spaces in the inter-rods (interprismatic), as depicted in Figure 6d, while the presence of interprismatic spaces is more discreet, as observed in Figure 6b,c.

## 4. Discussion

The daily practice of brushing aims to disorganize the biofilm formation [1,2], with the help of therapeutic agents (toothpastes) that can control dental dysbiosis [3] and/or help remineralize the dental substrate. However, brushing must not abrade the tooth surface, aggravating the irreversible loss of this structure. In this study, the impact of activated charcoal (powder or toothpaste) on enamel’s physico-mechanical properties and on the chemical aspects of slurries was evaluated. The results showed that there was a decrease in microhardness and an increase in roughness for activated charcoal toothpaste and toothpowder, rejecting the first hypothesis raised. Furthermore, these products did not alter the color (ΔE) of the teeth, rejecting the second hypothesis raised. It was also observed that the activated charcoal powder had a basic pH, did not contain fluoride and exhibited Ca^2+^ release compared to the other toothpastes, leading to the rejection of the third and fourth hypotheses.

The abrasiveness of toothpastes is due to the high concentrations of abrasive particles contained in toothpastes, such as silica [7], and in this study, only the activated charcoal toothpaste (AC-T) (Table 1) contains hydrated silica as an abrasive particle. The activated charcoal toothpowder used was derived from coconut shells, as specified by the manufacturer. Sujiono et al. [34], found that activated coconut shells contain fragmented charcoal microtubule particles with a predominance of longitudinal shape, with different sizes and shapes. The SEM images from this study, shown in Figure 5, confirmed the presence of an inhomogeneous structure within the activated charcoal powder, as reported in the literature [34,35]. Impurities such as hydrogen (H), oxygen (O), nitrogen (N), potassium (K), sodium (Na), phosphorus (P), calcium (Ca), and magnesium (Mg) can be found in the activated charcoal due to the presence of soil macronutrients naturally present in the coconut shell [34,35], which justifies the presence of some impurities detected by EDX in our study. As the study aimed to investigate tooth bleaching based on the removal of both external and internal pigments, the induction of staining was performed as a methodological tool to simulate clinical conditions and evaluate potential agents, variables, protocols and secondary effects of dental bleaching procedures [19,22]. To simulate brushing cycles, 3240 brush strokes at 0.6 Hz were performed in this study using a standardized brushing machine, which is consistent with previous studies [23,29]. Although, there is no consensus on the exact number of cycles, it has been reported that 824 strokes simulate 14 days of brushing [27], while approximately 30,000 brush strokes correspond to 2 years of manual toothbrushing [36,37]. Furthermore, 50,000, 75,000, and 100,000 brush strokes simulate 10, 15, and 20 years of brushing, respectively [9,38]. It is important to note that the degree of wear or surface alteration of the dental substrate is also influenced by the duration of brushing.

The results of the present study showed that brushing with charcoal toothpowder/toothpaste led to a decrease in microhardness and an increase in enamel roughness, which is consistent with systematic reviews indicating that activated charcoal has a high abrasive potential [10]. Some studies [23,36] did not show a significant increase in enamel roughness between the dentifrices tested, but they examined dentifrices with different compositions and experimental times. However, other studies with similar results to our findings showed that activated charcoal-based products decreased microhardness [14] and increased enamel surface roughness [14,26,27,39,40], and increased roughness decreased enamel gloss [9], which may contribute to increased microbial retention [41]. Therefore, factors such as particle composition, size, and type of particles [7] along with excessive brushing force, contribute to a higher degree of abrasion [17].

SEM micrographs showing the topography of brushed enamel have been reported in the literature at different magnifications [23,26], but these magnifications are generally smaller than those presented in our study, which sought to explore in more detail the effect that the use of these toothpowder/toothpaste causes to the tooth surface. At ×5000 magnification, the HP-T group showed more evident interprismatic spaces [Figure 6d], which is consistent with the significant changes in enamel roughness observed in this study. In this context, further investigations are needed to understand how the bleaching agent penetrates the enamel through these interprismatic spaces into the dentinal tubules. This suggests that H_2_O_2_ (hydrogen peroxide) likely passes through the enamel with minimal interaction with its organic matrix, resulting in a more homogeneous distribution [42]. Therefore, Müller-Heupt et al. [43], found that 6% hydrogen peroxide resulted in very mild interprismatic dissolution. The conventional dentifrice (C-T) which showed the least change in roughness also a showed smooth surface morphology pattern in the SEM. In contrast, both AC-P and AC-T showed greater visibility of enamel prisms at ×5000 magnification.

More irregular surfaces have been observed in SEM images after the use of activated charcoal-based products [14], which is in accordance with our findings. Thus, the variable size and fractal shape of the activated charcoal particles and the impurities present in the material make it potentially more abrasive, causing surface changes in the enamel. The relationship between particle shape and enamel abrasiveness has already been observed by previous studies [39,44], as the more direct contact of large AC particles, as opposed to the interaction of dentifrice with the tooth surface, may be associated with this finding [37]. It has been demonstrated that the abrasive wear rate increases linearly with the particle size and concentration until a critical size is reached [45]. No changes in enamel color were observed after brushing with either toothpowder or toothpaste (*p* = 0.676). There is insufficient evidence in the literature to support the effect of AC on altering tooth color [14,26,27,39,40], and even the whitening paste would not have sufficient concentration to promote a whitening effect. Supposedly, activated charcoal-based products would have their action based on their abrasive capacity and their ability to adsorb chromophores and extrinsic pigments from the tooth surface [12]. However, it is important to note that these products are only able to remove extrinsic stains, confirming the results of this study that all groups showed a relative change in ΔE, but no statistical difference between them. Therefore, removal of extrinsic pigments should not be confused with the effect of bleaching agents [23,40]. Similarly, changes in enamel roughness do not have a significant correlation with overall changes in tooth color, as roughness affects the brightness and the green-red axis of tooth color [46].

The toothpowder and toothpaste analyzed in this study had a pH above 7, representing neutral or basic slurries, confirming the literature [47]. Toothbrushing with acidic toothpastes has been shown to result in a slight increase in F^−^ concentrations in the biofilm fluid compared to neutral formulations, which may contribute to the greater anticaries effect of acidic formulations [31]. Some toothpowders exhibit an acidic pH, depending on the composition and micronutrients present in the raw materials used in their manufacture [34]. While it has been suggested that the pH of toothpaste slurries does not influence demineralization or the decrease in enamel microhardness, the greatest decrease in microhardness was observed when the enamel was treated with the non-fluoride toothpaste [47].

A chemical evaluation of the slurries showed that the toothpastes studied contained soluble fluoride (F^−^) in excess of 1000 ppm, with the exception of the toothpowder (AC-P), which did not contain any fluoride in its composition. The toothpowder (AC-P) also exhibited higher levels of free Ca^2+^ ions, which was observed after calculating the difference between the final and initial amount of Ca^2+^. Although Ca^2+^ was found in the initial analysis of AC-P group, likely due to impurities in the activated charcoal from coconut shells [34,35], it is also hypothesized that the high amount of Ca^2+^ found in the solution after brushing may be a by-product of tooth wear (Figure 4). As for the dentifrice used as a control (C-T), its composition indicates the presence of calcium carbonate (CaCO_3_), which confirms the presence of initial Ca^2+^ in our measurement and in the literature [32] but with no significant difference between the final (T1) and initial (T0) Ca^2+^ values (Figure 4).

The oral environment is constantly exposed to cariogenic/erosive challenges and the presence of F^−^ and Ca^2+^ ions during brushing could influence enamel remineralization [17]. Fluoride is the main agent indicated to control dental erosion with a minimum of 1000 ppm of chemically soluble F^−^ in toothpastes ensuring its preventive potential [4]. The presence of free ions after demineralization induces the precipitation of minerals on the tooth surface in the form of fluorapatite (FA). This mineral is less soluble than hydroxyapatite (HA) because its critical pH decreases from 5.5 to 4.5, which means that even if HA dissolves, the calcium and phosphate are replaced in the form of FA [48].

The addition of calcium and/or phosphate salts to fluoridated toothpastes has been proposed as a strategy to reduce the concentration of F^−^ in the products without compromising their anti-caries efficacy, thereby reducing the risk of dental fluorosis [32]. However, the calcium incorporated into toothpastes cannot be linked to an increased availability of F^−^ and Ca^2+^ in the biofilm fluid [32].

The clinical importance of this study lies in raising awareness about the importance of dental brushing but avoiding the risks associated with the indiscriminate use of activated charcoal powder containing large, irregular particles. The role of toothbrushing is the mechanical removal of biofilm [2], which is essential for maintaining oral health, and related to this is the therapeutic effect of toothbrushing by releasing remineralizing ions and fluoride (F^−^) into the environment. Based on the findings of our study, the use of charcoal toothpowder, which lacks fluoride (F^−^) or remineralizing ions/control of dysbiosis [16] could compromise the preventive action proposed in brushing. Furthermore, the mechanical action of charcoal toothpowder could potentially cause irreversible damage to the enamel surface. Controlling the particle size and quality of toothpowders, associated with the addition of remineralizing ions, could help to develop safer products for daily use.

## 5. Conclusions

The in vitro evaluation of toothbrushing with activated charcoal (powder or dentifrice) on the physical–mechanical properties and chemical aspects of slurries have been analyzed and discussed. The following conclusions can be drawn from this study:

(1) Brushing with CA-based toothpowder/toothpaste or whitening toothpaste did not induce any significant color change in the dental substrate.
(2) The enamel surface brushed with CA-based toothpowder/toothpaste showed an increase in Ra and a decrease in HK, highlighting the potential risks associated with indiscriminate prolonged use of these materials.
(3) The topographical morphology of the enamel showed less significant changes specifically when brushed with conventional dentifrices, because the action of the soft bristles of these dentifrices, combined with low force intensity, only acted on the hygienization of the tooth surface.
(4) The solutions resulting from brushing with the investigated toothpowders and toothpastes are alkaline. A deficiency of soluble fluoride and a higher release of calcium were observed after brushing with the activated charcoal toothpowder.

However, further long-term studies are needed to develop dental products that not only provide enhanced protection but also reduce the risk of enamel surface damage. Such products should prioritize the inclusion of ions capable of remineralizing or reducing the demineralization of dental structures, while promoting effective biofilm removal without causing abrasion of the dental surface.

## Figures and Tables

**Table 1:** Description of the products.

Groups (*n* = 12)	Whitening Agent	General Composition (Amount of Fluorine Provided by the Manufacturer)
AC-T	Activated charcoal-Toothpaste	Aqua, Glycerin, Hydrated Silica, Sodium Lauryl Sulfate, Aroma [Contains Mentha Piperita (Peppermint Oil)], Cellulose Gum, Xanthan Gum, Sodium Fluoride, Sodium Saccharin, Charcoal Powder, Benzyl Alcohol, Eugenol. Contains: Sodium Fluoride (1450 ppm F^−^)
AC-P	Activated charcoal-Powder	Coconut shell activated charcoal, Kaolin clay, Orange essential oil. No contains fluoride.
HP-T	Hydrogen Peroxide-Toothpaste	Propylene glycol, calcium pyrophosphate, glycerin, PEG/PPH/116 copolymer 66, PEG-12, PVP-hydrogen peroxide, PVP, silica, aroma, tetrasodium pyrophosphate, sodium lauryl sulfate, disodium pyrophosphate, sodium monofluorphosphate, saccharin sodium, sucralose, BHT, eugenol. Contains: Hydrogen peroxide 1%, sodium monofluorphosphate 0.76% (1000 ppm F^−^)
C-T	Control-Toothpaste	Calcium Carbonate, Aqua, Glycerin, Sodium Lauryl Sulfate, Sodium Monoflurophosphate, Cellulose Gum, Aroma, Tetrasodium Pyrophosphate, Sodium Bicarbonate, Benzyl Alcohol, Sodium Saccharin, Sodium Hydroxide. Contains: Sodium Monoflurophosphate (1450 ppm F^−^)

**Table 2:** Mean values and standard deviation (SD) of HK and Ra obtained for brushing protocols, before (T0) and after treatments (T1).

Groups	Microhardness (HK)			Roughness (Ra)		
	Before Brushing (T0)	After Brushing (T1)	*p* Value	Before Brushing (T0)	After Brushing (T1)	*p* Value
AC-T	231.69 (6.18) ^a^	193.66 (8.62) ^b^	0.001	0.111 (0.034) ^a^	0.202 (0.097) ^Bb^	0.011
AC-P	222.52 (2.08) ^a^	186.77 (8.87) ^b^	0.005	0.113 (0.036) ^a^	0.196 (0.090) ^Bb^	0.012
HP-T	227.33 (6.57) ^a^	230.91(6.14) ^a^	0.410	0.119 (0.034) ^a^	0.225 (0.116) ^Ab^	0.002
C-T	232.97 (6.19) ^a^	222.30 (5.67) ^a^	0.092	0.100 (0.032) ^a^	0.177 (0.073) ^Cb^	0.006
*p* value	0.069	0.001		0.183	<0.001	

Distinct lowercase letters differ statistically (*p* < 0.05) for each period of evaluation T0 and T1 (lines) and different capital letters differ statistically (*p* < 0.05) between types of toothpastes/brushing protocols (columns).

**Table 3:** Results for ΔE values (CIELAB color change), mean values and standard deviation (SD) of the experimental groups after the brushing protocol.

Experimental Groups	Mean ΔE (SD)	*p*
AC-T	4.55 (1.72)	0.676
AC-P	4.14 (2.7.2)	
HP-T	5.79 (1.63)	
C-T	5.97 (2.12)	

## Data Availability

Data are contained within the article.
